# News and Notes

**Published:** 1907-10

**Authors:** 


### NEWS AND NOTES.

Prof. Ernest H. Starling, M.D., F.R.C.P., Jodrell, professor of physiology at University College, London, has been awarded the Baly Gold Medal of the Royal College of Physicians of London.

The Baltimore Medical College has purchased a lot 64 by 167 feet with building, at a cost of $30,000. A building for laboratory purposes will be erected on this site.

The Seventh International Zoological Congress was held in Boston from Aug. 19th, to Aug. 24th.

The French Minister of the Interior has notified all the prefects throughout France that they must organize a "mothers' dispensary" or consultation for infants.

The Zeitschrift fur Urologie is one of the best of all the journals published on genito urinary and sexual diseases. The current issue contains 95 pages of reading matter upon these subjects which is ably edited under the management of Casper Frisch, Lohnstein, Oberlander, Posner, and Zuckerhandle. Abstracts are given of all the leading articles pertaining to this class of disorders.

The twentieth French surgical Congress which was convened at Paris October 7, is to discuss the influence of the Roentgen rays on malignant tumors: transplantation of nerves, muscles and tendons in treatment of paralysis, and tuberculosis and cancer in connection with accidents.

The woman's Medical College of Pennsylvania is planning to erect a new hospital building and operating theatre on the ground next to the college in Philadelphia. The hospital building is to be six stories in height and the operating theatre is to be a two story building.

The attorney general has announced that the medical statute does not require that the applicant for a license shall be a citizen of the United States. If he is a graduate of a duly authorized Japanese medical college, with the requisite four years course of study, and if he is otherwise qualified, a license shall be issued if he pass

A hospital for consumptives is to be built at the Tennessee State penitentiary, where there are said to be at present 45 patients afflicted with the disease.

The Atlanta Board of Health has elected Mr. G. H. Brandon to succeed Dr. C. F. Benson as president of the board, Dr. Benson having recently resigned. Dr. C. W. Strickler was elected vice-president in place of Mr. Brandon. Dr. W. B. Armstrong was elected to succeed Dr. Benson as a member from the scond ward.

The Medical and Chirugical Faculty of Maryland visited the Jamestown Exposition September 11 and 12. Scientific meetings were held on the boat on both evenings, and in Norfolk they were welcomed by a committee from the Norfolk Medical Society.

It is alleged that 50 per cent. of the school children of Ohio are physically defective. One of every two is said to be a sufferer from disorder of the vision, bad teeth, throat affections or other ailments.

The report of the Sanitary Department of the Isthmian Canal Commission shows a death rate among the white employees for June of 21.05 per thousand per annum; among the blacks of 29.96 per thousand.

The sixth International Dermatologic Congress was held at the Academy of Medicine, New York City, Sept. 9-14, 1907. An address of welcome on behalf of the United tates was delivered by Surgeon-General Rixey of the United States Navy; an address for the American Universities was delivered by Prof. Ira Remsen of John Hopkins University; and an address for the medical Profession of the United States by Dr. Joseph D. Bryant, President of the American Medical Association. Papers were read by some of the most famous European dermatologists.

---

The health officer of Cleveland, on account of the prevalence of barber's itch in that city, has had several barbers arrested because they do not follow the letter of the law as regards to cleanliness. Three were fined $5 and costs; two received suspended sentences, and the others asked to have their cases continued.

---

The Surgeon General of the United States Army has designated Capt. W. H. Moncrief to deliver a series of lectures on Tropical Diseases at the Atlanta School of Medicine during the coming winter. This important course will not be limited to students, but any physicians desiring to attend these lectures will be welcome. There will be a lecture a week during January and February. In connection with the above course access may be had to the surgeon General's library by those desiring to procure literature upon this subject.

---

Although Dowie renounced Physicians in public, when he was sick he quietly called one to attend him. Armstrong, the manager of the Christian Science Journal was also recently found sick with pleurisy and under the care of a regular physician.

---

E. B. Treat & Co. announce that they will soon have ready "'Clinical Treatises on the Symptomatology and Diagnosis of the Respiration and Circulation" by E. von Neusser, M. D. of Vienna, translated by A. McFarlane, M. D.

